# 纳米药物递送技术在克服肺癌耐药性中的应用

**DOI:** 10.3779/j.issn.1009-3419.2024.101.30

**Published:** 2024-11-20

**Authors:** Yingchun LU, Chunyu WANG, Bin LIU

**Affiliations:** ^1^610054 成都，电子科技大学医学院（鲁迎春）; ^1^School of Medicine, University of Electronic Science and Technology of China, Chengdu 610054, China; ^2^610032 成都，成都中医药大学医学与生命科学学院（王春语）; ^2^School of Medical and Life Sciences, Chengdu University of Traditional Chinese Medicine, Chengdu 610032, China; ^3^610042 成都，四川省肿瘤临床医学研究中心/四川省肿瘤医院·研究所/四川省癌症防治中心/电子科技大学附属肿瘤医院肿瘤内科（柳斌）; ^3^Department of Medical Oncology, Sichuan Clinical Research Center for Cancer, Sichuan Cancer Hospital & Institute, Sichuan Cancer Center, Affiliated Cancer Hospitalof University of Electronic Science and Technology of China, Chengdu 610042, China

**Keywords:** 肺肿瘤, 纳米药物递送技术, 耐药, Lung neoplasms, Nano-drug delivery technology, Drug resistance

## Abstract

肺癌是严重危害人类健康的恶性肿瘤之一，其死亡率在我国所有恶性肿瘤中位列第一。耐药的发生是导致肺癌患者生存结局不容乐观的重要因素，如何克服肺癌耐药性已成为亟待攻克的难题之一。纳米药物递送技术目前已成为克服肺癌耐药性的一种重要手段。针对肺癌发生耐药的机制，采用纳米药物递送技术可实现药物的联合递送、提高药物递送效率、增强药物靶向性和安全性等，为克服肺癌耐药提供了新思路。本文阐述了肺癌治疗的现状、发生耐药的机制及克服耐药性的策略，介绍了纳米技术在肺癌诊断和治疗过程中的应用，综述了纳米药物递送技术在克服肺癌耐药性应用中的最新研究进展，讨论了纳米药物递送技术的发展前景和目前面临的挑战。

## Abstract (en)

Lung cancer is one of the most malignant tumor, representing a significant threat to human health. In China, its mortality rate is the highest among all malignant tumors. The occurrence of drug resistance has resulted in unfavourable prognosis for patients with lung cancer, and overcoming drug resistance is a significant challenge that needs to be addressed. Nano-drug delivery technology has been an important approach to overcome drug resistance in lung cancer. Targeting to the mechanisms of drug resistance, by enabling the combined delivery of drugs, increasing the efficiency of drug delivery and improving the targeting and safety of drugs, nano-drug delivery technology offers a novel approach to tackling drug resistance in lung cancer. This paper describes the current status of lung cancer treatment, mechanisms of drug resistance, strategies to overcome drug resistance, and the application of nanotechnology in the diagnosis and treatment of lung cancer. In addition, it summarizes the recent research progress on the application of nano-drug delivery technology to overcome drug resistance in lung cancer. Finally, the current prospects and challenges of nano-drug delivery technology are discussed.

肺癌是一种全球常见的、严重危害人类健康的恶性肿瘤。据统计^[1]^，2022年全球新发肺癌病例及死亡病例均居首位，分别占恶性肿瘤病例总数的12.4%和18.7%。2022年中国癌症数据统计^[2]^显示，肺癌的死亡率在所有恶性肿瘤中位列第一。目前肺癌的治疗方案包括手术治疗、放疗、化疗、靶向治疗和免疫治疗，其中化疗是大部分肺癌患者治疗的主要选择，靶向治疗和免疫治疗药物的应用显著改善了部分患者的生存结局。由于早期筛查、诊断和治疗手段的共同进步，肺癌的死亡率逐年下降，但每年因肺癌造成的死亡人数仍然远远超过结直肠癌、乳腺癌和前列腺癌的总和^[3,4]^。耐药的发生是导致肺癌患者预后不容乐观的重要因素，亟待研究出对抗耐药的新方法。随着纳米医学的发展，纳米药物递送技术在癌症治疗领域崭露头角，其在改善药代动力学、精准靶向肿瘤、减少副作用和逆转耐药性等方面显示出巨大优势^[5]^。因此，目前除了采用联合应用不同药物、开发新的药物等方法克服肺癌耐药性以外，纳米药物递送技术的应用有望为克服肺癌耐药性提供新思路，本文将对纳米药物递送技术在克服肺癌耐药领域中的进展进行综述。

## 1 肺癌耐药机制及克服肺癌耐药性策略

肺癌分为小细胞肺癌（small cell lung cancer, SCLC）和非小细胞肺癌（non-small cell lung cancer, NSCLC），其中SCLC约占15%，NSCLC约占85%，NSCLC中又以肺腺癌最多见^[4]^。尽管近几十年来，靶向治疗和免疫治疗显著改善了晚期肺癌患者的生存结局，但是由于耐药性的发生，肺癌患者的预后不良^[6⇓-8]^。耐药是指肿瘤细胞产生多种防御机制以减少和抵抗药物的细胞毒性或抑制作用的能力。耐药的发生机制复杂，涉及原发性耐药、适应性耐药、肿瘤微环境（tumor microenvironment, TME）重塑、药理学限制及药物外排泵等^[9,10]^。肿瘤细胞由于预先存在的遗传或基因组耐药性变化，在初始治疗时便具有耐药性，即原发性耐药；还可通过激活适应性耐药程序如调控上皮间充质转化（epithelial-mesenchymal transition, EMT）及肿瘤干细胞（cancer stem cell, CSC）等以抵抗治疗^[9]^；此外，TME的重塑，如炎性微环境、缺氧状态、代谢重编程等也可促使肿瘤发生耐药^[11]^；同时，药理学限制及药物外排泵机制也是肿瘤发生耐药的重要因素，如介导药物外排的ATP结合盒（ATP-binding cassette, ABC）转运蛋白的过表达，可以促进药物外排并减少药物在细胞内的积累^[12]^。多种因素之间互相影响，促进耐药的发生发展。目前，克服肺癌耐药性的方法主要有3种^[9,13]^：（1）通过精准的个体化诊疗治愈早期肺癌以预防耐药的发生。针对高危人群的肺癌筛查有利于发现早期肺癌患者从而实现早诊早治；影像学和分子病理诊断技术的进步帮助临床快速明确肺癌病理类型、分期及分子分型以实施针对性的、个体化的治疗策略^[14]^。（2）根据基因突变或肿瘤耐药相关机制合成新的靶向药物。表皮生长因子受体-酪氨酸激酶抑制剂（epidermal growth factor receptor tyrosine kinase inhibitors, EGFR-TKIs）已普遍应用于治疗EGFR基因突变的肺腺癌患者，使用第一或第二代EGFR-TKIs后发生耐药的肺癌患者中约50%会出现EGFR T790M突变，针对这一突变位点，采用第三代EGFR-TKIs可有效克服耐药^[15,16]^。（3）联合用药以克服耐药。由于化疗、靶向治疗、免疫治疗等药物的作用机制不同，联合用药以靶向不同耐药机制可用于克服对单一药物的耐药，目前已有诸多临床研究^[17]^证明联合用药行之有效。

然而，目前传统的克服肺癌耐药的策略受到检测方法缺乏特异性或敏感性、药物缺乏靶向性、联合用药毒副作用显著增加等限制，如何有效克服肺癌耐药仍是亟待解决的重要难题^[9,18,19]^。纳米医学或许可以为这一难题提供解决方案。纳米材料是指在至少一个维度上其尺度为纳米级的结构，其独特的理化性质使之在靶向性、提高药物负载效率、药物共递送和多功能性等多个方面具有明显优势，其在肺癌的诊断和治疗中应用广泛，如影像成像、微创手术、药物递送、增强免疫应答、重塑TME等^[20⇓⇓-23]^。近年来，已有许多纳米药物进入了临床试验研究（表1），一些纳米药物如白蛋白结合型紫杉醇、多柔比星脂质体等已经被美国食品药品监督管理局（Food and Drug Administration, FDA）批准应用于临床。因此，基于上述纳米材料的优点及特性，以纳米材料作为载体，将药物递送到目标部位的纳米药物递送技术将是一种克服肺癌耐药性的有前途的新策略。

**表1:** 进入临床试验的用于肺癌治疗的纳米药物

Study title	Type of cancer	Status	Nanodrugs	Reference
Carbon nanoparticle-loaded iron [CNSI-Fe (II)] in the treatment of advanced solid tumor	Advanced solid tumor	Phase I	Carbon nanoparticle-loaded iron [CNSI-Fe (II)]	NCT06048367
Quaratusugene ozeplasmid (Reqorsa) and Atezolizumab maintenance therapy in ES-SCLC patients	ES-SCLC	Phase I Phase II	Reqorsa	NCT05703971
Quaratusugene ozeplasmid (Reqorsa) in combination with Pembrolizumab in previously treated NSCLC	NSCLC	Phase I Phase II	Reqorsa	NCT05062980
Quaratusugene ozeplasmid (Reqorsa) and Osimertinib in patients with advanced lung cancer who progressed on Osimertinib	NSCLC	Phase I Phase II	Reqorsa	NCT04486833
EP0057 in combination with Olaparib in relapsed advanced gastric cancer and SCLC	Gastric cancer; SCLC	Phase II	EP0057 (Nanoparticle camptothecin)	NCT05411679
Trial of EP0057, a nanoparticle Camptothecin with Olaparib in people with relapsed/refractory SCLC	Relapsed/refractory SCLC	Phase I Phase II	EP0057 (Nanoparticle camptothecin)	NCT02769962
Neoadjuvant LDRT combined with Durvalumab in potentially resectable stage III NSCLC	Stage III NSCLC	Phase I	Nanoparticle albumin bound paclitaxel	NCT05157542
Phase I/II randomized study of NBTXR3 activated by abscopal or radscopal radiation in combination with immunotherapy (anti-PD-1/PD-L1) for patients with advanced solid malignancies	Advanced malignant solid neoplasm	Phase I Phase II	NBTXR3 (Hafnium oxide-containing nanoparticles)	NCT05039632
NBTXR3 and radiation therapy for the treatment of inoperable recurrent NSCLC	Inoperable recurrent NSCLC	Phase I	NBTXR3 (Hafnium oxide-containing nanoparticles)	NCT04505267
Stereotactic brain-directed radiation with or without aguix gadolinium-based nanoparticles in brain metastases	Brain metastases	Phase II	AGuIX (Gadolinium-based nanoparticles)	NCT04899908
Nano-SMART: nanoparticles with MR guided SBRT in centrally located lung tumors and pancreatic cancer	NSCLC; Pancreatic cancer	Phase I Phase II	AGuIX (Gadolinium-based nanoparticles)	NCT04789486
Trial of NanoPac intratumoral injection in lung cancer	Lung cancer	Phase II	NanoPac (Sterile nanoparticulate paclitaxel)	NCT04314895
A randomized, double-blind, placebo controlled phase III study to investigate efficacy and safety of first-line treatment with HLX10+chemotherapy [Carboplatin-nanoparticle albumin bound (Nab) paclitaxel] in patients with stage IIIB/IIIC or IV NSCLC	Squamous NSCLC	Phase III	Nab Paclitaxel	NCT04033354
A study to test ABI-009 in patients with metastatic, unresectable, low or intermediate grade neuroendocrine tumors of the lung or gastroenteropancreatic system	Neuroendocrine tumors	Phase II	ABI-009 (Albumin-bound rapamycin Nanoparticles)	NCT03670030
A study of BIND-014 (Docetaxel nanoparticles for injectable suspension) as second-line therapy for patients with KRAS positive or squamous NSCLC	KRAS positive or squamous NSCLC	Phase II	BIND-014 (Docetaxel nanoparticles)	NCT02283320
Combination therapy with NC-6004 and gemcitabine in advanced solid tumors or NSCLC, biliary and bladder cancer	Solid tumors	Phase I Phase II	NC-6004 (Nanoparticle cisplatin)	NCT02240238
Paclitaxel albumin-stabilized nanoparticle formulation in treating patients with previously treated advanced NSCLC lung cancer	Recurrent NSCLC; Stage IV NSCLC	Phase II	Paclitaxel albumin-stabilized nanoparticle formulation	NCT01620190
TUSC2-nanoparticles and erlotinib in stage IV lung cancer	Lung cancer	Phase I Phase II	DOTAP (Cholesterol-TUSC2 liposome complex)	NCT01455389
A phase 2 study of CRLX101 (NLG207) in patients with advanced NSCLC	NSCLC	Phase II	CRLX101 (Nanoparticle formulation of camptothecin)	NCT01380769

Reference is referring to registration of trials in the clinical trials database (https://clinicaltrials.gov/). ES-SCLC: extensive stage-small cell lung cancer; LDRT: low dose radiation therapy; NSCLC: non-small cell lung cancer; PD-1: programmed cell death protein-1; PD-L1: programmed cell death ligand 1; MR: magnetic resonance; SBRT: stereotactic body radiation therapy; KRAS: Kirsten rat sarcoma viral oncogene homolog; TUSC2: tumor suppressor candidate 2.

## 2 纳米药物递送技术在克服肺癌耐药中的应用

纳米药物递送技术在新型药物的开发及实现联合用药等方面有显著优势，现已开发出了多种类型的纳米药物递送载体，包括脂质体、聚合物、无机纳米颗粒、量子点、外泌体、细菌纳米颗粒、核酸纳米颗粒等，可用于不同药物的递送^[24,25]^。目前已有许多关于纳米药物递送技术应用于克服肺癌耐药性的研究报道，如采用纳米药物递送技术检测相关生物标志物、改善影像成像效果，促进肺癌的早期准确诊断，指导早期肺癌的手术治疗^[9,26]^；借助纳米药物递送技术改善药物药代动力学特性，还可通过主动或被动靶向机制增强药物递送的靶向特异性，并可进行不同种类药物的联合递送，例如化疗药物、活性基因片段和免疫增强因子等，以达到增强疗效、减轻药物毒副作用等目的^[23,27⇓-29]^；另外，可根据纳米颗粒独特的理化性质设计被光、热、声、特殊的TME等特异性触发的纳米药物递送系统，实现可控性的精准治疗^[28,30⇓-32]^；还可以通过抑制外排转运蛋白而增加药物在细胞内的积累以提高药物治疗效果^[33]^。纳米药物递送技术在增强药物靶向性、实现联合用药及减轻药物毒副作用等方面具有显著优势，在克服肺癌耐药中有巨大的应用前景，目前已有许多关于纳米药物递送技术在克服肺癌对化疗、靶向治疗及免疫治疗耐药中的研究（表2^[28,34⇓⇓⇓⇓⇓⇓⇓⇓⇓⇓⇓⇓-47]^），进一步探索纳米药物递送技术将为肺癌耐药患者提供更多新的治疗方法。

**表2:** 用于克服肺癌耐药性的纳米药物递送技术

Nanostructure	Active principle (s)	Mechanism of action	Tumor model (s)	Reference
Nanomicelles	Paclitaxel+Triphenylphosphonium	Targeting mitochondrial; Enhancing drug uptake; Inhibiting P-glycoprotein	Cisplatin-resistant A549/ADR	[28]
Nanoparticles	Cisplatin prodrug+Triphenylphosphonium+lonidamine	Targeting mitochondrial; Inhibiting metabolic reprogramming	Cisplatin-resistant A549/DDP	[34]
Carbon nanotube	Carbon nanotube+Adriamycin	Enhancing intracellular drug internalization; Inhibiting ATP by mitochondrial damage	Adriamycin drug-resistant H69AR	[35]
Polymer-drug conjugates	Paclitaxel+Multidrug resistance1 siRNA	Inhibiting P-glycoprotein; Blocking autophagic flow	Taxol-resistant lung cancer cells A549/T	[36]
Nano-zinc carriers	Ubiquitin-specific protease (USP14)+Aptamer	Silencing USP14	Cisplatin-resistant A549/DDP	[37]
Formulated nano-liposome	Cisplatin+Nuclear protein zeste homolog 2-targeting peptide EIP103	Down-regulation of N-cadherin and vimentin proteins; Reducing the nerve growth factor receptor levels and increasing the peroxisome proliferator-activated receptor γ levels	Cisplatin-resistant A549/cis	[38]
Nanoparticles	Gefitinib+Thymoquinone	Retrieving MET phenomena	Gefitinib-resistant A549/GR	[39]
Dendritic-Polymer-Based Nanococktail	Gefitinib+Yes-associated protein siRNA	Blocking the EGFR signaling pathway; Inhibiting activation of the EGFR bypass signaling pathway; Inducing tumor cell apoptosis; Impairing glycolysis	Gefitinib-resistant NSCLC cells	[40]
Nanoparticles	Osimertinib+Selumetinib	Inhibiting both EGFR and mitogen-activated protein kinase	Osimertinib-resistant HCC827/AR	[41]
Supramolecular therapeutic nanoplatform	Gefitinib	Promoting the apoptosis of tumor cells; Inhibiting the activation of the bypass IGF1R signaling pathway	Gefitinib-resistant PC9-GR	[42]
Polymer modified mesoporous silica nanoparticle	Volasertib+PD-L1 antibody	Inhibitiing PLK1; Upregulating PD-L1 expression	KLN205	[43]
Copper sulfide nanocarriers	Epacadostat+Dasatinib	Regulating T cell-related immunosuppression	A549	[44]
Cationic liposomes	SGT-53	Altering the TME to increase immune cell infiltration and activation	LL/2	[45]
Nanoparticle	Polydopamine+Alpha-tocopherol succinate	Enhancing the mitochondrial targeting impact and promoting cell apoptosis by changing the mitochondrial membrane potential	A549； Lung Lewis carcinoma	[46]
Chitosan-PLGA-based nanoparticle	miR29A-B1+miR34A+CASP8	Targeting resistance to apoptosis	A549	[47]

ATP: adenosine triphosphate; siRNA: small interfering RNA; USP14: ubiquitin-specific protease 14; MET: mesenchymal to epithelial transition factor; EGFR: epidermal growth factor receptor; IGF1R: insulin-like growth factor 1; PLK1: Polo-like kinase 1; TME: tumor microenvironment; PLGA: poly(lactic-co-glycolic acid); CASP8: caspase 8.

### 2.1 纳米药物递送技术在克服肺癌化疗耐药中的应用

化疗是大部分肺癌患者的主要治疗选择，虽然大多数患者最初对治疗敏感，但容易发生耐药并发生疾病的快速进展^[7,48]^。因此，克服化疗耐药性对于改善患者临床预后有重要意义。通过纳米药物递送技术将药物靶向递送到肿瘤组织中可以有效提高药物疗效、减轻药物不良反应、克服耐药性^[27]^。Wang等^[28]^构建了一种靶向线粒体的紫杉醇（paclitaxel, PTX）纳米胶束，通过将PTX精准递送至线粒体，使得线粒体ATP水平降低，导致依赖于ATP的药物外排泵P糖蛋白（p-glycoprotein, P-gp）生物活性被抑制，相较于游离PTX，通过该纳米胶束靶向递送的PTX在肿瘤组织中的积累增加、抗肿瘤活性增强，有效逆转肿瘤细胞对PTX的耐药性，且未见明显全身毒性。Lu团队^[34]^设计合成了一种可靶向肿瘤细胞表面CD44的自组装纳米靶向给药系统，将顺铂精准递送至肿瘤细胞线粒体，损伤线粒体功能，克服肺癌细胞对顺铂的耐药性。有研究^[35]^发现由于碳纳米管（carbon nanotube, CNT）可以在肺部蓄积，基于此原理设计了一种将CNT与阿霉素（doxorubicin, DOX）共价结合的纳米药物，增加DOX在耐药SCLC细胞H69AR内的蓄积进而克服耐药。另外，使用纳米药物递送技术实现联合用药也可改善肺癌化疗耐药。Wang等^[36]^组装了一种纳米聚合物，可将PTX和沉默多重耐药蛋白1（multidrug resistance protein 1, MDR1）表达的小干扰RNA（small interfering RNA, siRNA）共同递送至肿瘤细胞，抑制MDR1基因编码的P-gp的表达，通过化疗联合基因治疗的策略克服肺癌对PTX的耐药性。同时，针对化疗耐药机制研究新型纳米药物也有助于改善化疗耐药，Zhao等^[37]^发现泛素化特异性蛋白酶14（ubiquitin-specific proteases 14, USP14）与肺癌患者对顺铂耐药有关，对顺铂耐药的肺癌细胞其USP14表达高于对顺铂敏感的肺癌细胞，通过适体纳米锌载体靶向递送USP14 siRNA可增强顺铂对耐药肺癌细胞和荷瘤小鼠的抗肿瘤作用。Zeste增强子同源物2（enhancer of zeste homolog 2, EZH2）的高表达与肿瘤对铂类的耐药密切相关，拮抗EZH2的表达有望提高肿瘤细胞对铂类药物的敏感性，基于此，Li等^[38]^开发了一种共递送EZH2拮抗剂及顺铂的脂质体纳米药物，结果显示该纳米药物可逆转NSCLC对顺铂的耐药，抑制肿瘤增殖与转移。纳米药物递送技术应用于克服肺癌化疗耐药领域具有广阔前景，其有望为因发生化疗耐药而预后不良的肺癌患者带来新的希望。

### 2.2 纳米药物递送技术在克服肺癌靶向治疗耐药中的应用

具有特定基因组突变的肿瘤患者可从分子靶向治疗中受益^[49,50]^，但这部分患者占比不到25%，并且在治疗过程中几乎普遍产生耐药性，耐药性的发生成为了癌症靶向治疗的最大障碍^[4,13,50,51]^。EMT是获得性耐药发生的重要因素，Priyanka Upadhyay团队^[39]^制备了一种转铁蛋白修饰的纳米颗粒NP-dual-3，与吉非替尼（Gefitinib, GEF）联合应用于对GEF耐药的A549细胞，发现NP-dual-3可以限制EMT，恢复耐药A549细胞对GEF的敏感性。开发纳米药物共递送系统也是克服肺癌靶向治疗耐药的重要方法之一。Yes相关蛋白（yes-associated protein, YAP）已被确定为EGFR-TKIs耐药发生的关键驱动因素，Huang等^[40]^通过采用纳米颗粒靶向共递送GEF和YAP-siRNA，阻断EGFR信号通路的同时，通过YAP-siRNA沉默YAP的表达以抑制EGFR旁路信号通路的激活，克服耐GEF的NSCLC细胞系异种移植物的耐药性。Chen等^[41]^开发了一种用于联合递送奥希替尼（Osimertinib, OSI）和司美替尼（Selumetinib, SEL）的活性氧（reactive oxygen species, ROS）响应性纳米颗粒，通过响应TME中高水平的ROS增强药物的靶向特异性并减少药物在正常组织中的积累，抑制EGFR信号通路和丝裂原活化蛋白激酶（mitogen-activated protein kinase, MAPK），克服了NSCLC细胞对OSI的耐药性，并且相较于游离药物的联合治疗，采用该纳米药物共递送技术在减轻联合用药的全身毒性方面具有一定优势。此外，有研究^[42]^设计了一种基于“AND”布尔逻辑的超分子药物递送系统，将光动力疗法（photodynamic therapy, PDT）与GEF靶向治疗相结合，通过药物的精准递送和严格的可控释放提高了治疗的时空特异性，增强疗效的同时减轻了药物副作用，在对EGFR-TKIs耐药的肺癌肿瘤模型中显示出良好的抗肿瘤效果。针对肺癌患者对靶向治疗耐药的机制，未来有望采用纳米药物递送技术实现个体化精准治疗，克服对靶向治疗的耐药性，改善患者预后。

### 2.3 纳米药物递送技术在克服肺癌免疫治疗耐药中的应用

免疫检查点抑制剂（immune checkpoint inhibitors, ICIs）治疗，已被确立为广泛期SCLC和无EGFR/ALK突变的局部晚期/转移性NSCLC患者的标准治疗^[48]^。ICIs的应用已使部分肺癌患者达到长期获益，但大多数患者由于免疫耐药等原因仍会出现疾病进展。免疫耐药的发生与癌基因和癌蛋白、抗原缺失、T细胞功能失调、TME、肿瘤免疫代谢紊乱以及遗传和表观遗传功能失调等有关^[6]^。目前克服免疫耐药的方法包括：阻断免疫共抑制信号、激活免疫共刺激信号、调节TME和靶向T细胞活化等^[52,53]^。调节TME是克服肺癌免疫治疗耐药的重要方法，Reda等^[43]^发现Polo样激酶1（Polo-like kinase 1, PLK1）的表达与免疫抑制性TME有关，可抑制免疫细胞浸润和抗肿瘤免疫，因此他们设计了一种同时递送PLK1抑制剂及程序性死亡配体1（programmed cell death ligand 1, PD-L1）抗体的介孔二氧化硅纳米颗粒，抑制PLK1的同时上调PD-L1水平，增强PD-L1抗体对肿瘤细胞的靶向性和抗肿瘤效应，在ICIs难治性小鼠肺肿瘤模型KLN-205中观察到了良好的抗肿瘤作用，并且该纳米颗粒显著降低了药物作用的有效剂量，具有良好的安全性。另外，T细胞与肿瘤免疫密切相关，有一项研究^[44]^认为，耗竭或功能性抑制调节性T细胞（regulatory T cells, Tregs）与免疫治疗相结合是一种有价值的方法，因此研究人员制备了一种封装艾卡哚司他（Epacadostat）和达沙替尼（Dasatinib）的纳米载体，其具有良好的生物相容性、生物可降解能力和稳定性，并且可有效延长艾卡哚司他的药物半衰期，最终通过抑制和逆转支持Tregs的免疫抑制而起到良好的抗肿瘤作用。此外，联合免疫治疗与基因治疗也是克服肺癌免疫耐药的有效策略，在Kim等^[45]^的研究中应用了一种其内封装野生型TP53基因质粒DNA的新型脂质体SGT-53，将p53基因疗法与抗程序性死亡受体1（programmed death 1, PD-1）疗法相结合，通过减少免疫抑制和恢复抗肿瘤免疫反应，恢复NSCLC小鼠对抗PD-1治疗的敏感性。肺癌发生免疫耐药机制复杂，目前关于纳米药物递送技术在克服肺癌免疫耐药中的研究主要落足于通过联合免疫治疗与化疗、靶向治疗、放疗等其他治疗方式来调节免疫抑制、增强抗肿瘤免疫以提高ICIs疗效，逆转肺癌耐药。

### 2.4 其他

针对肺癌耐药发生机制，基于纳米药物递送技术，目前还研究出了其他的对抗肺癌耐药的方法，如：靶向线粒体、靶向细胞凋亡抗性等。由于线粒体在细胞凋亡和耐药性中的中枢调节作用，Meng等^[46]^制备了一种靶向线粒体的纳米颗粒，以聚多巴胺作为光热剂，将α-生育酚琥珀酸酯（alpha-tocopherol succinate, α-TOS）作为化疗药物精确地递送至肿瘤细胞的线粒体，通过化学和光热协同疗法抑制肿瘤生长。细胞凋亡抗性是NSCLC耐药性和侵袭性表型的主要驱动因素，Chattopadhyay等^[47]^通过靶向细胞凋亡抗性开发了一种新的NSCLC基因治疗方法，采用纳米颗粒递送肿瘤抑制因子miR29A-B1和miR34A以及CASP8用于治疗NSCLC，有效诱导了A549的细胞凋亡。另外，通过纳米药物递送技术着眼于铁代谢及铜死亡等也是目前研究的热点^[54⇓-56]^。现已有许多基于纳米药物递送技术的用于克服肺癌耐药性的方法正在被研究，但未来能否进一步实现临床转化仍有待探索。

## 3 纳米药物递送技术目前面临的挑战

基于纳米材料独特的理化性质，纳米药物递送技术在改善药物药代动力学、靶向递送药物、联合递送药物及控制药物释放等方面具有显著优势^[9,22,57]^，但其可能在安全性、生物相容性、全身毒性等方面面临巨大挑战。如搭载siRNA的纳米药物，可以在基因水平上靶向作用于肿瘤细胞，但高效递送和脱靶效应风险仍是需要解决的问题^[25]^。另外，纳米药物载体应用时可以与生物系统的各种成分相互作用，这些相互作用将可能导致一些无法预料的不良反应的发生^[58]^。并且纳米药物制备工艺相对复杂、制备成本高，如何在现有的技术条件下制备出可应用于临床的药物仍是一个难题。目前大部分纳米药物仍停留在临床前研究阶段，为探索纳米药物递送技术的安全性、有效性、毒副反应等，还需要更多的实验及临床数据支撑。除了受限于纳米材料本身安全性、生物相容性及制作工艺和成本等问题，将纳米药物递送技术应用于临床还面临以下挑战^[5]^，如：肺癌发生耐药的机制目前仍不明晰，复杂的TME对纳米药物释放过程中的精准调节存在巨大影响；临床前研究和临床试验之间存在差异，如何合理构建用于纳米药物体内抗肿瘤治疗研究的动物模型以评估临床试验前药物的效果及安全性；肿瘤异质性使得肺癌患者之间存在个体差异，临床应用过程中应如何对患者进行个性化的评估和筛选以选取最佳治疗方案等。

## 4 总结和展望

目前关于纳米药物递送技术克服肺癌耐药的研究大多局限于单一的诊断或治疗，不能评估疾病的动态变化过程，因此，综合多学科领域，针对肺癌耐药机制研究开发具有诊断、治疗与监测一体化功能的个性化纳米药物递送平台，通过早期诊断及在治疗过程中实时、动态评估治疗效果或许将是未来探索纳米药物递送技术用于克服肺癌耐药的一大方向^[59,60]^。另外，人工智能在药物的开发与优化、可控式的药物递送、数据分析整合、生物技术和生物系统模拟等多个方面具有显著优势，将人工智能与纳米药物递送技术相结合也是未来值得研究的方向之一^[61,62]^。尽管纳米药物递送技术目前仍存在许多问题，大部分关于纳米药物递送技术的研究还未实际应用于临床，但其独特的优势及发展的潜力不可忽略。将纳米药物递送技术真正应用于临床克服肺癌耐药的治疗是一条艰辛但充满希望的道路，值得我们去深入探索，以期未来实现更多的临床应用，为克服肿瘤耐药性、提高临床疗效提供新的治疗策略，实现个体化、精准化诊疗。
